# Practical considerations for the use of mTOR inhibitors

**DOI:** 10.1186/s13737-015-0029-5

**Published:** 2015-12-22

**Authors:** Fritz Diekmann, Josep M. Campistol

**Affiliations:** Department of Nephrology and Kidney Transplantation, Hospital Clínic, Villarroel, 170, E-08036 Barcelona, Spain; Servicio de Nefrología - Clinical Institute of Nephrology and Urology, Hospital Clinic, Barcelona, Spain

**Keywords:** Calcineurin inhibitors, mTOR-inhibitors, Nephrotoxicity, Post-transplant malignancy, Kidney transplantation

## Abstract

Immunosuppressive therapy after kidney transplantation is based on calcineurin inhibitors (CNI). In most cases CNI therapy is combined with mycophenolate and steroids. In spite of good short-term results this therapy is associated with long-term toxicities, graft loss and patient death. Therefore, alternative immunosuppressive strategies are needed that combine excellent efficacy with low incidences of long-term adverse outcomes.

This review focuses on the strategies based on mTOR- inhibitors in combination with minimized exposure to CNI.

## Introduction

Modern immunosuppressive regimens after kidney transplantation are associated with very low acute rejection rates during the first year and excellent 1-year graft and patient survival. Usually, 1-year acute rejection rates are observed to be between 5 and 15 % in a therapy based on calcineurin-inhibitors (CNI) and mycophenolate mofetil or mycophenolate sodium (MMF/MPA) [1]. One-year patient survival can be expected to be above 90 % as well as 1-year graft survival. The above-mentioned immunosuppressive regimens based on a CNI and MMF, however, are associated with considerable toxicities and other drug-related adverse events such as direct nephrotoxicity, worsening of several cardiovascular risk factors and a higher incidence of cardiovascular events compared with the general population, a higher incidence of post-transplant cancer as well as a high incidence of viral infections such as CMV, BKV and oncogenic viruses. Many of these adverse events can be attributed to CNIs directly or the combination of a CNI with MMF. Moreover, many of these adverse effects are CNI-dose-dependent. During the last 30 years the search for an alternative to the potent CNIs has not yielded a treatment that is routinely employed. Belatacept-based therapy might be an alternative in patients with low to moderate immunological risk [2], however, it has not yet acquired an important market share, partly due to its high cost and partly due to concerns about less immunosuppressive potency. The dilemma of current immunosuppressive therapy is as thus: the need to use a drug for its potency in the absence of an equivalent alternative but one which is associated with toxicities that favor the development of complications or risk factors that can ultimately lead to the death of the patient such as hypertension, nephrotoxicity, viral infections, diabetes mellitus and cancer. In fact, the main causes of death of kidney allograft recipients beyond 1 year post-transplant are fatal cardiovascular events and post-transplant malignancy [3]. This dilemma has led to the development of immunosuppressive strategies that CNIs and maintain a sufficient long-term immunosuppressive potency. These regimens – based on just the necessary exposure to CNIs with potent concomitant immunosuppressive medication – reflect the current international guidelines recommending the combination of a CNI (preferably tacrolimus [TAC]) and MMF [4]. This therapy has led to the best results in terms of graft and patient survival so far but these results are still not satisfactory, since the main causes of death are still cardiovascular events and cancer, and the main reasons for long-term graft loss are antibody-mediated damage and chronic rejection [5]. In other words, further minimizing CNI exposure in a CNI/ MMF combination will result in more immunological damage long-term while incrementing the CNI exposure will result in a higher incidence of CNI-associated adverse events. Therefore, several strategies that allow potent immunosuppression and low toxicities have been developed. This review will focus on strategies based on mTOR-inhibitors, and especially based on sirolimus.

### mTOR-inhibitor-based CNI avoidance de novo and early conversion

#### mTOR-based CNI avoidance de novo

The quest for avoiding CNI exposure has led to several studies of complete CNI avoidance de novo [1, 6]. However, only one monocenter study comparing an mTOR-inhibitor-based conversion with induction with an IL2R-antibody with a CNI-based regimen, yielded satisfactory results in favor of the mTOR-inhibitor [7]. Other multicenter studies with a comparable regimen showed higher acute rejection rates in the mTOR-inhibitor-based arms [1, 6]. An alternative would be to add induction with a lymphocyte-depleting antibody to yield better efficacy. Lebranchu et al. conducted a study comparing cyclosporine A (CsA), MMF and a steroid regimen with sirolimus, MMF and steroid regimen. Patients in both arms received induction treatment with anti-thymocyte globulin. Efficacy, graft and patient survival as well as renal function at 1 year were comparable in both arms [8]. However, in general mTOR-inhibitor-based CNI-free de novo regimens have been associated with a high incidence of side effects and have therefore not found their way into clinical routine treatment.

#### mTOR-inhibitor-based early conversion

Lebranchu et al. performed a study of early conversion from CsA-based treatment to sirolimus at 3 months post-transplantation in combination with MMF and oral steroids, with planned discontinuation at month 8 [9]. A total of 192 of 237 patients were converted. Cockcroft-Gault-clearance at 1 year was significantly better in the sirolimus group (68.9 vs. 64.4 mL/min). Patient and graft survival were not statistically different. The incidence of acute rejection episodes, mainly occurring after withdrawal of steroids, was numerically, but not statistically higher in the sirolimus group (17 % vs. 8 %, *p* = 0.071). Sixteen patients discontinued sirolimus, mainly for adverse events (*n* = 11), and seven patients discontinued CsA for renal failure or acute rejection. Significantly more patients in the sirolimus group reported aphthous ulcers, diarrhea, acne and high triglyceride levels.

Budde et al. performed a similar trial of early conversion from Cs A to everolimus 4.5 months after transplantation [10]. Five hundred and three patients were included, however only 60 % of these patients could be randomized to receive everolimus, or continue standard CsA-based treatment. Patients in both arms received MMF. Everolimus patients experienced a significant improvement in glomerular filtration rate (GFR) (71.8 mL/min vs 61.9 mL/min). However, rates of biopsy-proven acute rejection were higher in the everolimus group after conversion (10 % vs 3 %). Recently published 5-year confirmed the 1 year results with an overall higher rate of acute rejection, but significantly better kidney allograft function [11].

Weir and colleagues evaluated early CNI withdrawal and introduction of sirolimus in combination with MMF [12]. After 1 year, the mean percentage change from baseline in measured GFR was significantly higher in the MMF/sirolimus group compared with the MMF/CNI group, however, at 2 years, the change was indistinguishable. Calculated creatinine clearance and GFR were significantly greater with MMF/sirolimus at 2 years. Biopsy-proven acute rejection occurred in 14 MMF/sirolimus-treated patients (3 graft losses) and in 17 receiving MMF/CNI (6 graft losses). No patients died receiving MMF/sirolimus, but five treated with MMF/CNI did.

Conversion patients participating in the ZEUS trial who were evaluated in a separate analysis showed a higher incidence of de novo donor-specific antibodies [13]. Most of these patients had also the steroids withdrawn. The observed phenomenon by Liefeldt *et al* might possibly reflect under-immunosuppression rather than a genuine drug effect.

Moreover, in the above-mentioned conversion trials more patients were withdrawn from the mTOR-inhibitor-based treatment due to adverse events.

In general, early CNI withdrawal with subsequent mTOR-inhibitor-based treatment is a valid strategy for a select group of patients, however, it should not be offered to patients with moderate-to-high immunological risk as it is associated with a higher incidence of adverse events.

#### Practical considerations for conversion from a calcineurin inhibitor to an mTOR- inhibitor

Planned or reactive conversion strategies have yielded a variety of results depending on the drug combinations, patient selection and timing of the conversion. Reactive conversion from a CNI to an mTOR-inhibitor is safe in terms of graft and patient survival and acute rejection rates, however, it does not result in significant improvement of graft function [14]. Early or very early conversion may be associated with a higher rate of adverse events [15, 16]. All conversion studies share common drawbacks. A certain number of patients cannot or do not want to be converted for various reasons. A planned conversion usually takes place in a situation when the patient is asymptomatic, and psychologically, this might not be an ideal moment to convince a patient of the necessity of a new treatment with potential new side effects. Consequently, a higher number of adverse events results in a higher number of withdrawals in the conversion patients [9, 10, 15].

Therefore, for many patients it might be reasonable to receive a de novo treatment that is also used later on as the maintenance treatment.

In spite of this the following situations might give rise to a reactive conversion to SRL:

- CNI-associated cosmetic side effects
- BKV or CMV infection
- Papilloma virus infections
- Skin cancer and solid organ other cancers
- CNI-associated neurotoxicity

#### Minimization of calcineurin inhibitors in combination with mTOR- inhibitors

CNIs are the mainstay of immunosuppression after kidney transplantation and rejection rates as low as 7 % during the first year have been achieved [2]. No standard immunosuppressive regimen free of CNI has been associated with such low rejection rates. In contrast, post-transplant malignancy and CNI nephrotoxicity have both been associated in a dose-dependent manner with CNI. mTOR-inhibitors offer a superb opportunity to minimize CNI exposure and thus reduce potential hazardous CNI-associated side effects. The combination of mTOR-inhibitors and low-dose tacrolimus is probably the most potent immunosuppressive regimen after kidney transplantation in the era of modern immunosuppression.

mTOR- inhibitors were first used in combination with CsA in the second half of the 1990s and, at that time, resulted in remarkably low acute rejection rates without induction therapy. However, two major drawbacks were associated with this therapeutic approach: CNI and mTOR-inhibitors at full doses are associated with a number of severe side effects and secondly mTOR-inhibitors increased the nephrotoxicity of CsA. Consequently, different dosing strategies were developed including a reduction in the mTOR-inhibitor dose used, since most of the mTOR-inhibitor-associated side effects are dose-dependent. Furthermore, due to the immunosuppressive potency of the combination, CNI exposure could be drastically lowered.

Clinical data on the use of mTOR-inhibitors in combination with low-dose tacrolimus are limited. Langer et al. showed that treatment with everolimus  allowed early and substantial tacrolimus minimization when used with basiliximab induction and corticosteroids [17]. In this study 234 patients were randomized at month four to receive very low-dose tacrolimus treatment (2–4 ng/mL) or low-dose tacrolimus (4–7 ng/mL) in combination with everolimus and steroids. During the first 3 months, patients received low-dose tacrolimus in combination with everolimus and steroid treatment as well as basiliximab induction. At 1 year, acute rejection rates were comparable between cohorts. In the study arm a tacrolimus concentration of 3.4 ng/mL was achieved. This study demonstrated the feasibility of extremely low tacrolimus doses in combination with an mTOR-inhibitor.

Sirolimus has also been used successfully in combination with low-dose tacrolimus, resulting in comparable efficacy and less nephrotoxicity when compared with sirolimus in combination with standard dose tacrolimus.

Russ and colleagues studied 64 patients who received sirolimus in combination with reduced dose or standard dose tacrolimus. Patient and graft survival as well as the incidence of acute rejection were no different between the groups with different tacrolimus exposure. Moreover, reduced tacrolimus exposure combined with sirolimus showed a trend towards better renal function while simultaneously maintaining comparable acute rejection rates [18].

Peddi *et al*. reviewed CNI minimization therapy in combination with an mTOR-inhibitor [19]. They identified and evaluated 21 relevant studies and concluded that immunosuppressive regimens including an mTOR-inhibitor and tacrolimus minimization better preserve renal function versus standard-dose tacrolimus, without significant changes in patient survival or graft rejection rates. Rates of infection and malignancies were low. Other adverse events were more commonly reported including dyslipidemia/hyperlipidemia in up to two thirds of patients, new-onset diabetes mellitus in up to 38 %, wound complications in up to 22 %, and hypertension in up to 17 %.

#### Sirolimus dosing in combination with tacrolimus

Kahan et al. found that renal transplant recipients treated with sirolimus and CsA achieved sufficient protection from acute rejection with sirolimus concentrations > 5 ng/mL [20], whereas an acceptable rate of adverse events was achieved with concentrations < 15 ng/mL. So far, a similar assessment for the combination of sirolimus and tacrolimus has not been performed. However, data from the ASSET trial [17] showed that mTOR-inhibitor concentrations of > 3 ng/mL in combination with a CNI offer sufficient protection from rejection.

Adverse effects of mTOR-inhibitors appear to be dose-related [20]. Therefore, sirolimus drug levels need to be carefully monitored. Monitoring of sirolimus trough concentrations and careful evaluation of possible adverse reactions is the key to avoiding side effects and improving side effect management. Loading doses of sirolimus should be avoided.

Sirolimus dose requirements vary with the type of CNI used. Ciancio and colleagues demonstrated that sirolimus exposure is lower in combination with TAC than with CsA [21]. Therefore, higher sirolimus doses are needed in combination with tacrolimus compared with the sirolimus/ CsA combination.

## Conclusions

mTOR-inhibitors can be used as non-nephrotoxic drugs in order to minimize CNI exposure. This drug combination has enabled dramatic CNI dose reductions in the early post-transplant phase. Both CNI and mTOR-inhibitors have a narrow therapeutic window and a high risk of side effects. Moreover, they are characterized by high intraindividual and interindividual differences of dosing requirements. Therefore, therapeutic drug monitoring of both drugs are necessary. A summary of the different studies of use of the mTOR-inhibitors with and without CNIs either de novo or in early conversion is given in Tables 1 and 2. Possible indications and disadvantages are summarized in Table 3.

**Table 1:** Overview of studies with sirolimus

Author	Study arm	Control arm	Outcome
Ekberg et al. [1]	Dac + SRL low + MMF + S (a)	CsA + MMF + S (d)	Higher AR in SRL arm (a. 37 %, b 24 %, c 12 %, d 26 %)
	Dac + CsA low + MMF + S (b)		
	Dac + Tac low + MMF + S (c)		
Flechner et al. [6]	Dac + SRL + MMF + S (e)	Dac + Tac + MMF + S (g)	Higher AR in SRL/MMF (e 31 %, f 15 %, g 8 %)
	Dac + SRL + Tac WD + S (f)		
Flechner et al. [7]	Dac + SRL + MMF + S	Dac + CsA + MMF + S	Better GFR in SRL arm (67 vs. 51 mL/min)
Lebranchu et al. [8]	Thy + SRL + MMF + S	Thy + CsA + MMF + S	Better GFR in SRL arm (54 vs. 45 mL/min)
Lebranchu et al. [9]	Dac + CsA + MMF + S early conversion to SRL	Dac + CsA + MMF + S	Better GFR in SRL arm (69 vs. 64 mL/min)
c [12]	CNI + MMF+/−S (+/− induction) early conversion to SRL	CNI + MMF+/−S (+/− induction)	No difference in GFR change at 2 yrs
Guba et al. [15]	ATG + CsA + MMF + S very early conversion	ATG + CsA + MMF + S	Better GFR in SRL arm (65 vs. 53 mL/min)

*Dac* Daclizumab, *SRL* Sirolimus, *MMF* Mycophenolate mofetil, *S* steroids, *CsA* Cyclosporine A, *Tac* Tacrolimus, *AR* acute rejection rate, *WD* withdrawal, *GFR* glomerular filtration rate, *Thy* Thymoglobuline®, *CNI* Calcineurin inhibitor, *ATG* ATG Fresenius®

**Table 2:** Overview of studies with everolimus

Author	Study arm	Control arm	Outcome
Budde et al. [10, 11]	Bas + CsA + MPS + S early conversion to EVR	Bas + CsA + MPS + S	Better GFR EVR arm at 1 yr and at 5 yrs (72 vs. 62 mL/min at 1 yr); overall higher rate of AR
Mjörnstedt et al. [16]	CsA + MPS + S early conversion to EVR (7 weeks)	CsA + MPS + S	GFR change 4.9 vs 0 mL/min (EVR vs CsA)
Langer et al. [17]	Bas + Tac low + EVR + S + conversion Tac very low	Bas + Tac low + EVR + S	No difference in AR post-conversion; GFR 57 vs. 51 mL/min (*p* = 0.0299)

*S* steroids, *CsA* Cyclosporine A, *Tac* Tacrolimus, *AR* acute rejection rate, *WD* withdrawal, *GFR* glomerular filtration rate, *Bas* Basiliximab, *MPS* Mycophenolate Sodium, *EVR* Everolimus

**Table 3:** Possible indications and disadvantages

Treatment regimen	Patients who might benefit	Disadvantages
De novo mTOR-I-based without CNI	- Low immunological risk patients	- Wound-healing problems in obese patients
	- CNI-related side effects to be anticipated (neurotoxicity, nephrotoxicity, CNI-associated hemolytic uremic syndrome)	- High incidence of side effects of mTOR-I and mycophenolate combination
	- Patients at risk of CMV or BKV infection	- Probably induction with lymphocyte-depleting antibodies necessary
	- Patients at risk of post-transplant malignancy	
De novo mTOR-I and CNI combination	- Low and intermediate immunological risk patients	- Wound-healing problems in obese patients
	- Patients who do not tolerate an adequate dose of mycophenolate	- Possible risk of new-onset diabetes
	- Patients at risk of CMV or BKV infection	
	- Patients at risk of post-transplant malignancy	
